# Tuina combined with diet and exercise for simple obesity: A protocol for systematic review

**DOI:** 10.1097/MD.0000000000028833

**Published:** 2022-02-11

**Authors:** Lili Chen, Deyu Cong, Gaofeng Wang, Jiabao Sun, Yuanyuan Ji, Zhen Zhong, Tong Liu, Jiayi Liu, Yunjie Chu, Xingquan Wu

**Affiliations:** a Department of Acupuncture and Tuina, Changchun University of Chinese Medicine, Changchun, China; b The Affiliated Hospital of Changchun University of Chinese Medicine, Changchun, China; c Beihua University, Jilin, China.

**Keywords:** diet and exercise, protocol, simple obesity, systematic review, Tuina

## Abstract

**Background:**

The incidence of simple obesity is increasing annually, with the number of obese people in all age groups increasing significantly. Obesity has become an important public health concern. Simple obesity affects not only appearance but also health. Obesity has an increasing impact on individuals, families, and society. Therefore, the treatment of obesity is becoming increasingly important. Prior studies have shown that Tuina combined with diet and exercise is capable of producing improvements in body weight and fasted health markers. In recent years, there are many clinical studies on the intervention of simple obesity by Tuina combined with diet and exercise, however, no study systematically evaluated the clinical efficacy. The purpose of this study is to evaluate its effects of Tuina combined with diet and exercise on people with simple obesity.

**Methods:**

We will search the following electronic databases: PubMed, EMBASE, MEDLINE, Web of science, Cochrane Library, WanFang Data, CBM, CNKI, and VIP from the inception of the coverage of these databases to December 2021. Randomized controlled clinical trials related to Tuina combined with diet and exercise intervention on simple obesity will be included. Cochrane's collaboration tool will be used to assess the quality of the studies. RevMan 5.3 software will be used for the data analysis.

**Results:**

This study will provide a standardized evaluation for the efficacy of Tuina combined with diet and exercise for simple obesity.

**Conclusion:**

The conclusion of this study will provide evidence for the safety and effectiveness of Tuina combined with diet and exercise on weight loss.

**Ethics and dissemination:**

Ethical approval is not required for systematic review and meta- analysis. The results of this review will be disseminated in a peer-review journal.

**PROSPERO registration number:**

INPLASY202210079.

## 1 Introduction

### 1.1 Description of the condition

In recent years, obesity has become a global health problem.^[1]^ About one-third of the population is identified as obese or overweight in our world.^[2]^ Simple obesity refers to the abnormal accumulation of fat in the body, resulting in excess body weight (BW) without obvious disease. Studies have shown that,^[3,4]^ simple obesity is related to many factors, such as diet, physical inactivity, environmental endocrine disruptor bisphenol A(BPA) pollution, social psychological factors, etc. Meanwhile, simple obesity is also a risk factor for other complications, such as diabetes, cardiovascular and cerebrovascular diseases, and so on.^[5]^ In the era of rapid development of medical technology, the diagnosis and treatment of obesity should be targeted intervention according to individual differences of patients.^[6]^

Small degree of weight loss can also bring benefits to the body for obese patients, so clinical guidelines suggest that we should have lifestyle interventions for obese patients.^[7]^ Studies have shown that high-sugar and high-fat diet can stimulate the reward center of the brain to further influence the obesity degree of patients.^[8]^ So, it also includes some dietary adjustment methods. The combination of diet, exercise, and other lifestyles can reduce weight and bring benefits to obese patients.^[9,10]^ Health promotion, nutrition education, healthy life motivation, self-health management ability, and other actions are effective measures to weight loss.^[11–13]^ Tuina therapy has achieved good curative effect in the clinical treatment of simple obesity.^[14]^ Combining diet and exercise with Tuina therapy, continuous intervention is of great significance for obesity patients to lose weight and prevent complications.

### 1.2 Description and function of intervention

Tuina therapy, as one of the external treatment of Traditional Chinese medicine, can be performed on the abdomen, limbs, and other parts of the human body, it is safe and effective. Tuina has a better effect to weight loss for obese patients, it through the stimulate on the skin, meridians, acupoints, and other parts of the human body, the use of the method such as kneading, vibration, rubbing, and other techniques. Manipulation is applied to the specific part of the body surface to regulate physiological and pathological conditions of the body, so as to dredge the channels and collaterals, regulate Qi and blood, dispel pathogenic factors and strengthen health, and harmonize Yin and Yang. At the same time, patients also need to have a certain sense of self-management. Diet and exercise is the main means of disease popularization. Relevant studies have confirmed that multimode health education can be explained through multiple channels and appropriate education methods, it can be selected for different individuals to improve the cooperation between patients and medical staff. Different diet and exercise management methods play an important role in the intervention of simple obesity.^[15–18]^

Nowadays, the combination of online and offline health education has been realized through multimedia education, telephone interviews, offline lectures, and other ways. Obesity related health education content includes the cognition of disease, diet structure, physical exercise, regular work and rest, psychological intervention, and so on. At present, the popularity of electronic products, sedentary office environment and other factors, a series of social and economic disadvantages, and unhealthy lifestyle can all cause weight gain or obesity.^[19]^ In short, we can help patients and their families understand that the purpose of treating obesity and the harm of obesity through health education. Help them establish a healthy lifestyle to further improve their quality of life, and avoid the harm brought by blind weight loss. Due to the different research environment and research methods, there is still a lack of systematic sorting of the research in this field.

### 1.3 Why the review is important

Obesity is a common metabolic and endocrine disease which seriously affects patients’ quality of life, and long-term physical and mental health.^[20]^ Currently, some drugs such as (Naltrexone-bupropion [Contrave], Orlistat [Xenical, Alli], Saxenda, and Qsymia)^[21,22]^ are used to treat obesity, it still has some limitations owing to considerable drug-related side effects (e.g., headache, dizziness, insomnia, constipation, and gastrointestinal adverse effects), and it is not widely used in many areas. For the same reason, there are risks associated with surgery. Overall, these findings suggest that Tuina has a positive impact on both BW and blood lipids management. It has become more and more popular with patients and doctors based on its unique advantages of effectiveness, simplicity, and convenience. However, there are no studies on the effect of a combined application of Tuina plus lifestyle treatment in patients with simple obesity. In conclusion, it is necessary to evaluate the effectiveness and safety of Tuina combined with diet and exercise for obese patients.

## 2 Methods

The systematic review will be performed following the guidelines of the Preferred Reporting Items for Systematic Review and Meta-Analysis Protocols (PRISMA-P) 2015.^[23]^ Our protocol has been registered in PROSPERO register of systematic review network (No. INPLASY202210079). All steps of this systematic review will be performed according to the Cochrane Handbook (5.3.0).

### 2.1 Selection criteria

#### 2.1.1 Types of studies

We will include the randomized controlled clinical trials in this study. Published reports on the efficacy and clinical trials of Tuina combined with diet and exercise in the treatment of simple obesity are also included. Randomized controlled trials (RCTs) that involve at least 1 Tuina combined with diet and exercise related treatment to simple obesity, and 1 control treatment will be included. The studies involving non-RCTs, animal experiments, reviews, and case series will be excluded.

#### 2.1.2 Types of patients

Patients diagnosed with simple obesity, an age between 18 and 60, will be included in this study. There is no limitation on the sex, ethnicity, cultural background, and territory. Secondary obesity and gestational obesity will be excluded.

#### 2.1.3 Types of interventions and comparisons

The intervention measures of Tuina therapy for simple obesity, which includes traditional Chinese Tuina, acupoint Tuina, therapeutic Tuina, full body Tuina, and so on. The propaganda and education of diet campaign includes 2 ways online and offline, such as public account push, small program supervision and reminder, etc. The research objects are not limited to students, workers, middle-aged and elderly people in the community, grassroots and other obese people. The main contents of education cover the cognition of obesity and its hazards, the arrangement of daily diet structure, personalized exercise, etc. Studies on Tuina combined with diet and exercise therapy combined with drugs, surgery, and other therapies will be excluded. The intervention type of control group is diet and exercise therapy.

### 2.2 Types of outcome measure

The primary outcome will be total effective rate, body mass index (BMI), BW, waist circumference (WC). BMI, which combines height and weight to determine obesity, is the most important indicator for the diagnosis and assessment of obesity. WHO defines obesity as the BMI ≥28 kg/m^2^ in Asian people and ≥30 kg/m^2^ in non-Asian people. WC ≥90 cm for male and 80 cm for female. WC is the main index of abdominal obesity. And then the age and gender obesity-related indicators of the included population are evaluated for statistical analysis, course of disease, education background, quality of life questionnaire score, self-satisfaction score by Body-Esteem Scale.

### 2.3 Exclusion criteria

The following situations will be excluded:

(1) the data reported are insufficient to establish the results (e.g., participant size measures and Standard Deviation are insufficient);
(2) the data are duplicated or not extracted;
(3) the full article is not available.

### 2.4 Search strategy

In order to evaluate the efficacy of Tuina combined with diet and exercise in the treatment of simple obesity, we will search PubMed, EMBASE, MEDLINE, Web of Science, Cochrane Library, WanFang Data, CBM, CNKI, and VIP databases. We will collect RCTs published from inception to December 2021, regardless of language or form. The databases will be searched by combining the subject words with random words. The retrieval strategy is shown in Table 1 using PubMed retrieval as an example. The search terms were adapted appropriately to conform to different syntax rules of different databases.

**Table 1:** Retrieval strategy of PubMed.

Number	Search term
#1	“Massage therapy” [MeSH] OR “massage” [Title/Abstract]OR “anmo” [Title/Abstract] OR“acupressure”[Title/Abstract]OR“tuina”[Title/Abstract]OR“manipulate”[Title/Abstract]
#2	“Simpleobesity”[Title/Abstract] OR “overweight” [Title/Abstract] OR “fat” [Title/Abstract]
#3	“Diets” [MeSH] OR “diet” [Title/Abstract]OR “Caloric Reducing” [Title/Abstract] OR “fasting” [Title/Abstract]
#4	“Exercises” [MeSH] OR “Exercise Training” [Title/Abstract]OR “Physical Activity” [Title/Abstract]OR“Activities”[Title/Abstract]OR“AerobicExercises”[Title/Abstract]
#5	“Randomized controlled trial”[Title/Abstract]OR“Controlled clinical trial” [Title/Abstract]
#6	#1 AND #2 AND#3AND#4 AND #5

### 2.5 Data collection and analysis

#### 2.5.1 Selection of data

**Figure 1:**
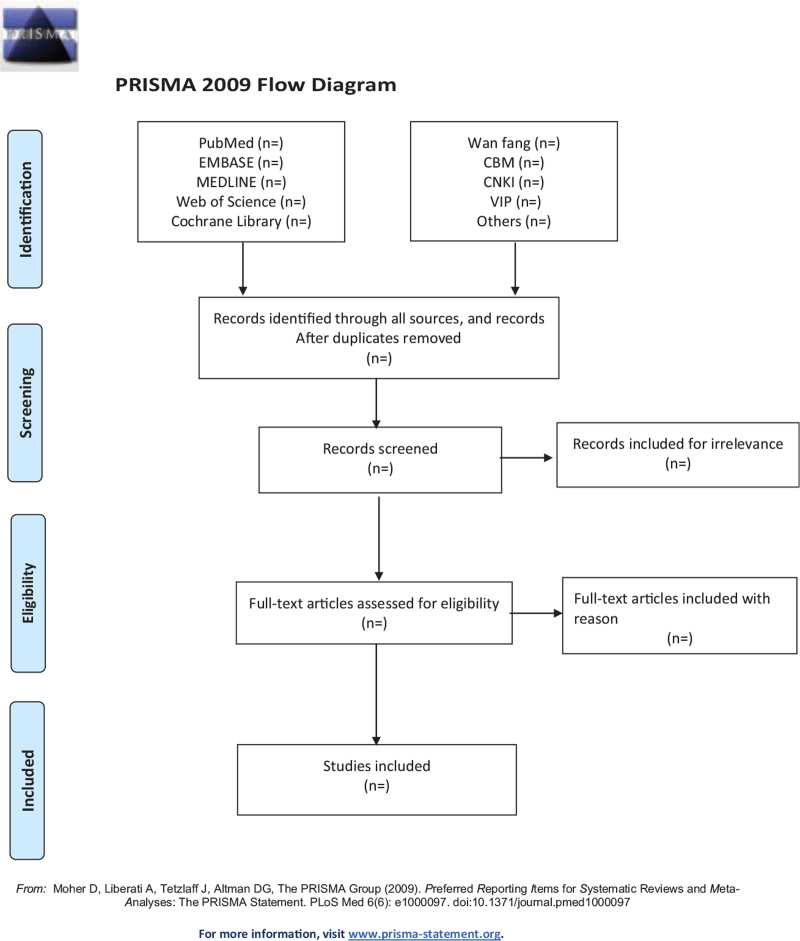
Flow diagram of literature retrieval.

Two authors will retrieve all the documents we need in the database based on the correct subject terms, import these documents into the document manager EndNote software V. X9.0 (Thomson ResearchSoft, Stanford, America), and delete the duplicate documents. Then read the titles and abstracts of the remaining documents, and delete the documents irrelevant to this systematic review. If the reading of the abstract and title cannot determine whether the document meets the standard, the researcher will read the full text of the article before making a judgment. Finally, download the remaining articles one by one to read the full text, and then determine the final required documents according to the various standards discussed above. In this process, the 2 researchers need to operate completely independently and strictly follow the operating procedures of the systematic review. If 2 people have ambiguity about the same article, ask the third reviewer to negotiate. If the full literatures are unable to obtain or related data are incomplete, we will contact the corresponding author. Third-party experts will be consulted to determine the selection divergence. The whole process of study selection is summarized in the PRISMA flow diagram. Screening operation will be rendered in Figure 1.

#### 2.5.2 Data extraction

According to the data extraction table of the Cochrane Handbook, the 2 researchers will extract the following data respectively: general information (national journal of first author publication year, etc); number of participants (gender, age, etc); intervention (Tuina type, diet, exercise type, treatment time and location, etc) compared with intervention (treatment type, course of treatment, etc); outcomes of included studies (BMI, BW, waist-hip ratios, etc) and adverse reactions. When 2 reviewers had differences in the data extraction process, the third author was responsible for solving them.

#### 2.5.3 Risk of bias assessment

Two researchers will evaluate the quality of the included studies under the guidance of the Cochrane Collaboration tool RevMan software version 5.3 (The Cochrane Collaboration, Oxford, England) for RCTs. The 2 reviewers will complete this work independently. If there is any disagreement, they can discuss and resolve it, and the third researcher can be involved when necessary. The Cochrane Collaboration tool assesses the following 7 domains: generation and allocation of random sequences, blinding of participants and personnel, blinding of outcome assessor, incomplete outcome data, selective reporting, and other risk biases. According to the above evaluation items contained in the literature, we will designate the “unclear risk of bias” or “low risk of bias” or “high risk of bias” to provide a better understanding of the included study quality.

#### 2.5.4 Data synthesis

When meta-analysis is permitted, statistical analysis will be performed using the Cochrane Review Manager (RevMan 5.3) software. Binary data represent risk ratios, and continuous data represent the average difference between different trials when results are measured in the same way. A 95% confidence interval will be used as the effective size for the joint analysis.

#### 2.5.5 Assessment of heterogeneity

I^2^ will be used to assess the statistical heterogeneity among trails. I^2^ > 50% indicates that the evidence is heterogeneous, while I^2^ < 50% will be taken as the combined results of no heterogeneity. If the *P* value exceeds .1 and I^2^ is less than 50%, the fixed effects model will be applied. A random effect model will be used when *P* value is less than .1 and I^2^ is over 50%.

#### 2.5.6 Analysis of subgroups

If the condition allows, we will perform a subgroup analysis. The following subgroup analyses will be considered.

1. Gender of the patients.
2. Process of the patients.
3. Different types of Tuina therapies.

#### 2.5.7 Sensitivity analysis

When sufficient data are available, sensitivity analysis will be performed to test the robustness of the primary outcomes, which includes assessing the quality of the methods, the quality of the studies, and the impact of sample size and missing data.

#### 2.5.8 Assessment of reporting biases

The results of the meta-analysis will be presented in the form of a forest. If the studies included in meta-analysis are more than 10, funnel plot will be used to evaluate potential publication bias.

#### 2.5.9 Confidence in cumulative evidence

The level of evidence on outcomes will be assessed utilizing the Grading of Recommendations Assessment, Development and Evaluation. Based on this grading systems, the result will be categorized as high, moderate, low, and very low quality.

## 3 Discussion

Obesity is a global public health issue, which results in many health complications. Recent studies have found that maternal nutrition in early pregnancy is related to obesity in children and adults,^[24]^ and high BMI in 7-year-old children is related to overweight in adults.^[25]^ Therefore, intervention obesity should start as early as possible. With the gradual integration of big data and the medical field, ICT will also gradually become the right-hand man of clinical staff.^[26]^ Obesity management can be improved through the use of a grading system that includes health management centers, comprehensive lifestyle interventions and medical treatment, enhanced obesity education and training, and the use of advanced e-health technologies. As a complementary and alternative traditional Chinese medicine therapy, Chinese Tuina massage, also called Tuina in China, is an ancient traditional Chinese medicine treatment method. As a safe, effective, economical, and simple intervention, Tuina is more acceptable to the public than drugs. Tuina combined with diet and exercise plays a better role of weight loss. In recent years, there have been more and more clinical reports on the treatment of simple obesity, but there are limitations to assess the body composition of obesity,^[27]^ and high-quality follow-up studies are still insufficient. Therefore, we intend to conduct a meta-analysis of the effectiveness of Tuina combined with diet and exercise in treating simple obesity, in order to provide high-quality evidence and guidance to clinicians as well as researchers.

## Author contributions

**Conceptualization:** Lili Chen, Deyu Cong, Xingquan Wu.

**Data curation:** Yuanyuan Ji, Zhen Zhong, Yunjie Chu.

**Formal analysis:** Lili Chen, Deyu Cong, Gaofeng Wang, Xingquan Wu.

**Methodology:** Lili Chen, Deyu Cong, Jiabao Sun, Xingquan Wu.

**Software:** Tong Liu, Jiayi Liu, Yunjie Chu.

**Supervision:** Deyu Cong, Xingquan Wu.

**Writing – original draft:** Lili Chen.

**Writing – review & editing:** Deyu Cong, Xingquan Wu.
